# Ultrasound-Mediated Local Drug and Gene Delivery Using Nanocarriers

**DOI:** 10.1155/2014/963891

**Published:** 2014-08-17

**Authors:** Qiu-Lan Zhou, Zhi-Yi Chen, Yi-Xiang Wang, Feng Yang, Yan Lin, Yang-Ying Liao

**Affiliations:** ^1^Department of Ultrasound Medicine, Laboratory of Ultrasound Molecular Imaging, The Third Affiliated Hospital of Guangzhou Medical University, Guangzhou 510150, China; ^2^Department of Imaging and Interventional Radiology, Prince of Wales Hospital, The Chinese University of Hong Kong, Shatin, New Territories, Hong Kong

## Abstract

With the development of nanotechnology, nanocarriers have been increasingly used for curative drug/gene delivery. Various nanocarriers are being introduced and assessed, such as polymer nanoparticles, liposomes, and micelles. As a novel theranostic system, nanocarriers hold great promise for ultrasound molecular imaging, targeted drug/gene delivery, and therapy. Nanocarriers, with the properties of smaller particle size, and long circulation time, would be advantageous in diagnostic and therapeutic applications. Nanocarriers can pass through blood capillary walls and cell membrane walls to deliver drugs. The mechanisms of interaction between ultrasound and nanocarriers are not clearly understood, which may be related to cavitation, mechanical effects, thermal effects, and so forth. These effects may induce transient membrane permeabilization (sonoporation) on a single cell level, cell death, and disruption of tissue structure, ensuring noninvasive, targeted, and efficient drug/gene delivery and therapy. The system has been used in various tissues and organs (in vitro or in vivo), including tumor tissues, kidney, cardiac, skeletal muscle, and vascular smooth muscle. In this review, we explore the research progress and application of ultrasound-mediated local drug/gene delivery with nanocarriers.

## 1. Introduction

Drug resistance is a main obstacle for curative cancer chemotherapy. Therefore, strategies need to be developed to overcome chemotherapy resistance [1]. In recent years, tumor-targeted therapy has been appearing as a promising therapeutic choice for cancer treatment. The potential approach is to develop particular carriers which can facilitate the release of the payload locally in tissue by internal or external stimuli (such as heat, light, ultrasound, etc.). Tumor imaging should be performed before and during the external stimuli or treatment. The biodistribution of drug carriers is monitored by imaging, so that the optimal timing for the application of external stimuli can be achieved [2]. Nanotechnology has the potential to influence the detection, prevention, and treatment of cancer.

Microbubbles are commonly used as intravascular ultrasound imaging probes and are becoming increasingly popular tools for targeted drug delivery. However, the microsized particles could only stay in blood circulation and penetrate poorly into tumor tissues, so that the wide application of the particles for in vivo tumor therapy is limited [3]. Strategies have been advised that nanoparticles can be used to deliver drug/gene to targeted tissues [4]. Nanoparticle, used as a drug/gene delivery vehicle, can not only target specific cells and tissues, but also retain the biological activity of the drug/gene during transport. Ultrasound is a noninvasive and visual theranostic modality that can be used to track drug carriers, trigger drug release, and improve local drug sediment with high spatial precision [5, 6]. Therefore, the development of novel visible ultrasonic responsive nanosized drug/gene carriers is necessary.

## 2. Nanocarriers in Ultrasonic Therapeutic System

Nanoparticles have been widely used as nanocarriers in recent years. The family of pharmaceutical nanocarriers includes polymeric nanoparticles, nanoemulsions, liposomes, and micelles. Liquid emulsions and solid nanoparticles are used with ultrasound to deliver genes in vitro and in vivo. The small packaging allows nanoparticles to extravasate into tumor tissues. Ultrasonic drug and gene delivery from nanocarriers have tremendous potential because of the wide variety of drugs and genes that could be delivered to targeted tissues by fairly noninvasive means [7].

### 2.1. Properties of Nanocarriers

Nanocarriers, with the properties of smaller particle size and long circulation time, would be advantageous in diagnostic and therapeutic applications. They can pass through blood capillary walls and cell membrane walls to deliver drugs [8], thereby reducing the side effect and enhancing the curative effect of cancer therapy [9]. Furthermore, as targeted delivery carriers, gene/drug-loaded nanocarriers can release their associated payload upon insonation. Besides, nanocarriers decorated with targeting moiety can adhere to targeted tissues, which can promote intracellular uptake of drug delivery vehicles. Although the system of ultrasound-mediated drug delivery with nanocarriers has many advantages, there are still many challenges. On one hand, the nanocarriers should be small enough to travel freely in blood circulation. On the other hand, it should be large enough to prevent from renal excretion but stable enough to prevent the content from biodegradation until activated by ultrasound. Above all, the vehicle should control the release of drug/gene at the right time and right point [10].

### 2.2. Enhanced Permeability and Retention (EPR) Effect

The combined use of ultrasound and DNA-bound bubbles has been found to improve DNA transfection both in vitro and in vivo experiments compared with administration of naked DNA alone [11, 12]. Nanocarriers can be designed to avoid extravasation to normal tissues and recognition by cells of the reticuloendothelial system (RES), thereby extending circulation time in blood. This in turn permits passive targeting of nanocarriers. Passive targeting based on the EPR effect allows extravasation of nanoparticles through deficient tumor capillaries characterized by large inter-endothelial junctions [13, 14]. The pore cutoff size range between 380 and 780 nm has been seen in a large number of tumors [15]. Moreover, poorly lymphatic drainage of tumor can prolong retention of particles in tumor tissue. Besides, nanoparticles coated with polymer chains can protect blood protein from adsorption and particle from recognition by RES cells. Kirpotin et al. [16] shown the EPR effect was a possible mechanism for drug delivery to tumor tissues in vivo, but rather antibody-dependent binding or endocytosis.

### 2.3. Nanocarriers Designed for Ultrasound-Mediated Drug/Gene Delivery

**Figure 1:**
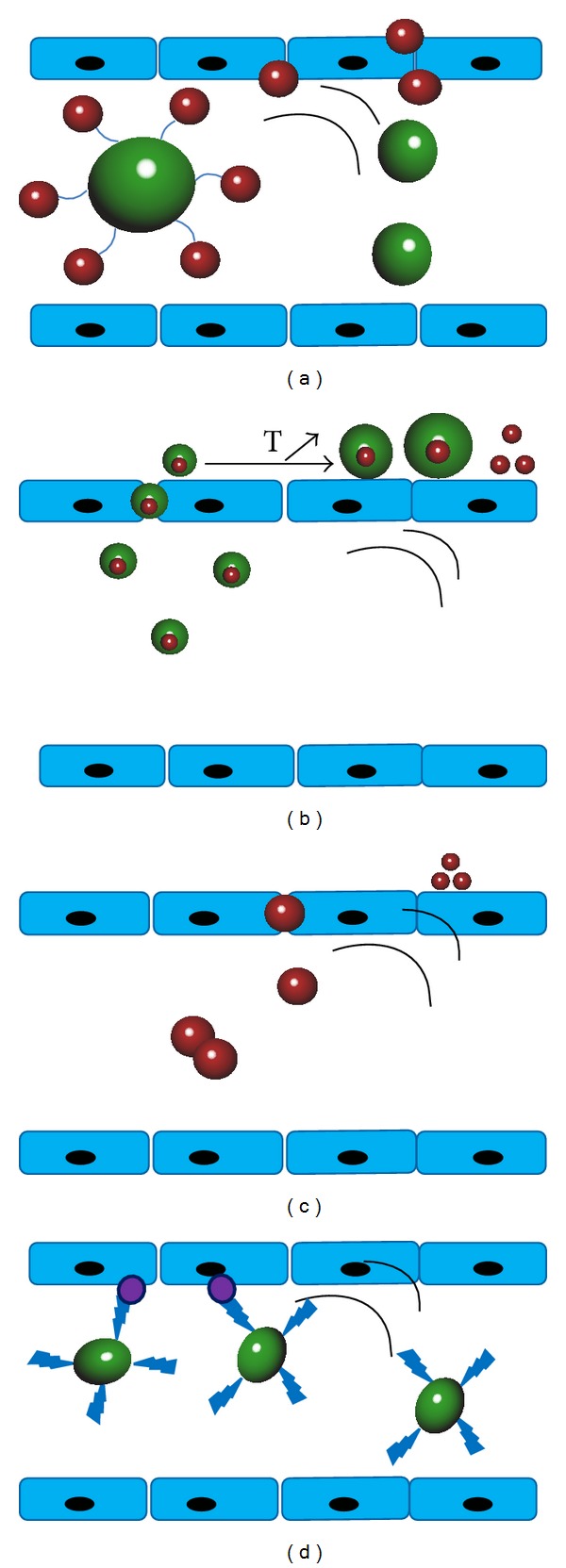
Schematic overview of various nano/microbubbles used for ultrasound-mediated drug/gene delivery. (a) The drug-loaded nano/microbubbles releasing drugs upon insonation. (b) Nanodroplets extravasate because of EPR and come into being microbubbles after a phase transition. (c) Nanosized lipospheres which can be activated by ultrasound in tumor tissues. (d) Bubbles associated with targeting moiety can adhere to the target molecules in tissue which express epitopes [17].

Some ultrasound contrast agent for ultrasound imaging is nowadays used as promising drug carrier, such as nanobubble. Since ultrasound is only applied at a certain location, time- and space-controlled drug delivery may become feasible. A straightforward strategy to load the bubbles with drugs is associating them with the superficial shell or even with its building blocks. Another way of loading is by encapsulating the drug into an oil reservoir presented in the core of the bubble. In addition, drugs can also be packed into nanoparticles that are subsequently attached to the microbubble's surface. As represented in following figure, four types of bubbles have been conceived for ultrasound-mediated drug delivery: (a) drug-loaded bubbles; (b) in situ formed nanodroplets; (c) acoustically active nanobubbles; (d) targeted bubbles (Figure 1) [17].

## 3. The Mechanisms of Ultrasound-Mediated Drug/Gene Delivery

The exact mechanisms of ultrasound-mediated drug/gene delivery with nanocarriers are still uncertain. According to the reports, they may be related to nonthermal effect (such as cavitation and mechanical effect) and thermal effect.

### 3.1. Nonthermal Effects

Nonthermal effects can be divided into cavitation and other mechanical effects [18]. Studies have shown that the combination of ultrasound and bubbles can increase the targeted delivery efficacy in vivo. The bioeffect may be attributed to the acoustic cavitation [19, 20]. Cavitation refers to the bubble activities induced by ultrasound, which can occur in liquid, liquid-like material containing bubbles and pockets containing gas or vapor. Under the action of adequately high ultrasonic pressure levels, the bubble oscillates and finally collapses. Cavitation can induce temperature rise, mechanical stress, and free radical production, thus influencing the biological function. The behavior of bubbles in low-intensity ultrasound field is different from high-intensity ultrasound field. Low-intensity ultrasound produces stable cavitation state, which can lead to intense friction and shear stress on the surrounding structures. When bubbles encounter high-intensity ultrasound (>1 MPa, 1 MHz), the amplitude of bubble oscillation rises instantly. The transient cavitation is produced, which can result in shockwaves and microjets [21]. Microjets can be described as a powerful stream of liquid caused by asymmetric implosion of microbubbles [22]. The microstreams give rise to temporary pores on surrounding vessel walls and cell membranes, promoting gene and drug targeted delivery [22–24]. Indeed, sonoporation (transient hole), induced by acoustic cavitation near the cell surface, has been shown to enhance the intracellular delivery of both small molecules and macromolecules [25–28]. Husseini and Pitt [7] reported that ultrasonic drug delivery from micelles usually employs polyether block copolymers and has been found effective in vivo for treating tumors. Ultrasound releases drug from micelles, most probably via shear stress and shock waves from the collapse of cavitation bubbles. It is also supposed that the release originates from acoustic streaming produced by radiation force. The collision of carriers may lead to shear stress, which results in reversible destabilization of the carrier and release of compounds. With the help of HIFU, drug releases from polymer micelles, which is most likely due to the effect of shear stress and/or shock waves produced by the collapse of a larger number of cavitation bubbles [29].

### 3.2. Thermal Effect

Another potential mechanism for ultrasound-mediated drug/gene delivery is a localized temperature rise in tissue. The temperature rise affects the liquidity of phospholipid bilayer, which directly results in changed membrane permeability. Ultrasound is used to trigger the collapse of cavitation bubbles, and the amplitude of the wave can produce high local temperatures. The main mechanism in the current therapeutic applications of ultrasound is creation of a controlled, localized temperature increase in situ [18]. This can cause hyperthermia, which is also known to increase the cellular uptake of anticancer drugs [30]. The possibility to achieve hyperthermia in situ through HIFU presents distinct improvements over conventional methods of heat generation in tissue. HIFU-induced hyperthermia has already been shown to produce significant enhancement of delivery of anticancer agents into tumor sites in vivo, with targeted release from thermosensitive liposomes [31, 32]. The combination of MR-guided focused ultrasound and drug-encapsulated nanocarriers could increase cellular uptake of agents [33].

### 3.3. Other Mechanisms

In fact, the mechanisms of ultrasound-mediated drug/gene delivery with nanocarriers may be associated with many other factors, such as endocytosis and active membrane transport. Targeted nanocarriers may change or fuse the phospholipid bilayer, so that lipid carriers release the payload contents directly into the cells [34]. Compared with equivalent thermal dose, pulsed-HIFU treatment leads to much enhancement in distribution of nanoparticles. Additional studies also proved that the effects enhanced by pulsed-HIFU sustained longer time than that of cavitation effect and heat, which offered another possible mechanism for ultrasound-mediated delivery [33]. Duvshani-Eshet et al. [35] suggested that therapeutic ultrasound by itself operated as a mechanical force which could drive the gene through the cell membrane and traversed from the cytoplasmic network to the nucleus, rather than by increasing membrane permeability. Transfection studies and confocal analyses showed that the actin fibers impeded transfection by ultrasound in BHK cells, but not in fibroblasts. A unique mechanism of drug delivery is supposed based on a so-called contact facilitated delivery, by which the phospholipid membranes of nanodroplets are merged into cell membranes of target cells, thus directly releasing their payload into the cytoplasm.

## 4. Commonly Used Nanocarriers for Ultrasound-Mediated Delivery

Various nanocarriers are being introduced and assessed, including organic and inorganic materials. Studies have reported that nanocarriers include polymeric nanoparticles, nanoemulsions, liposomes, and micelles. Recently, there have also been many inorganic materials used as nanocarriers, such as, metal nanoparticles, silica-based nanovehicles, and carbon-based nanovehicles [1]. Here we will mainly introduce the following several types and the research progress and application of the combination of ultrasound and nanocarriers for drug/gene delivery.

### 4.1. Polymeric Nanoparticles

Polymeric nanoparticles include nanospheres, nanocapsules, and polymersomes [36]. The most widely used polymers consist of poly(lactic acid) (PLA), poly(e-caprolactone) (PCL), and poly(lactic-co-glycolic acid) (PLGA) [37]. The polymer carriers used for the drug/gene delivery show properties of enhanced encapsulation and controlled release of contents in vitro [38]. Moreover, compared with natural polymers, synthetic polymers show higher purity and greater reproducibility. Polymers can be modified according to different requirements. For example, the polymeric nanoparticles copolymerized with polyethylene glycol (PEG) can avoid recognition by mononuclear phagocytic cells [39]. The polymeric shell also improves stability of the nanoparticles and increases their ability to withstand ultrasound pressure fields [40].

However, there are still many problems that may have an impact on the properties of the nanocapsules, such as the larger size [38]. Research progress with new ideas brings hope as well as many requirements to nanocapsules. The hybrid compounds prepared by the use of a metal and/or the active ingredients bring about great progress, such as research on the nanoparticle-based theranostic agents (nanoparticles which have both diagnostic and therapeutic functions). The commonly used metals include gold, iron, silver, and gadolinium. Such theranostic agents can be used for cancer diagnose and magnetic resonance imaging (MRI). In the case, iron oxide nanoparticles can encapsulate active ingredients to give the advantages of therapy and diagnosis.

Néstor et al. [40] prepared air-filled nanocapsules with a biodegradable shell consisting of PLGA. The nanocapsules were acquired by a modification of the double-emulsion solvent evaporation method. It had a mean size of 370 ± 96 nm and showed a high stability. The echogenic power in vitro provided an enhancement of up to 15 dB at a concentration of 0.045 mg/ML (at a frequency of 10 MHz). The signal loss for air-filled nanocapsules was 2 dB half an hour later. Yang et al. [41] developed a new type of US-triggered biodegradable nanocapsule, which was filled up with perfluorohexane (PFH), and the shell was formed by the DOX-loaded polymethylacrylic acid (PMAA) with disulfide crosslinking. The PMAA-PFH nanocapsules were very uniform, soft, and small (with a size of about 300 nm), which could easily enter the tumor tissues via EPR effects. The PMAA shell had high DOX-loading content (36 wt%) and great drug loading efficiency (93.5%), and the loading drug could be quickly released (<5 min) upon ultrasonic irradiation. The PFH filled could effectively enhance US imaging signal through acoustic droplet vaporization. What is more, the disulfide-crosslinked PMAA shell was biodegradable and thus safe for normal organisms. These merits enabled us optimize the balance of diagnostic, therapeutic, and biodegradable functionalities in a multifunctional theranostic nanoplatform.

Polymersome, as a nanocarrier, has been prepared for drug/gene delivery and therapy. Polymersome is a sort of synthetic vesicle, which is made of amphiphilic block copolymers and form a vesicle membrane that recalls the structure of lipids in cell membranes [42]. The amphiphilic block copolymers and polymersome are widely used for drug delivery systems due to the self-assembling ability in aqueous solutions [43]. Polymersome is a promising artificial vesicle, which has a large compartment, giving the characteristics of stability, an adjustable membrane, and the encapsulation of bifunctional compounds (hydrophilic and lipophilic molecules). Compared with liposomal formulation, the polymersome showed EPR effect and high-efficiency loading which was significant for the controlled release of drugs against tumors [44]. Yang et al. [45] developed a paclitaxel-loaded PEGylated immunoliposome with a particle size of 200 nm by post-insertion method, as a local drug delivery carrier, which showed high cellular uptake efficiency in rats.

Recently, smart polymer vesicles have attracted increasing interest due to their endless potential applications such as tunable delivery vehicles for the treatment of degenerative diseases. Chen and Du  [46] designed a novel polymer vesicle based on the PEO-b-P (DEA-stat-TMA) block copolymer, which was sensitive to both ultrasound radiation and pH in vitro. The dually responsive vesicle had no cytotoxicity less than 250 mg/mL and could encapsulate drugs efficiently, showing good release rate under the condition of ultrasound or lower pH.

### 4.2. Nanobubbles

**Figure 2:**
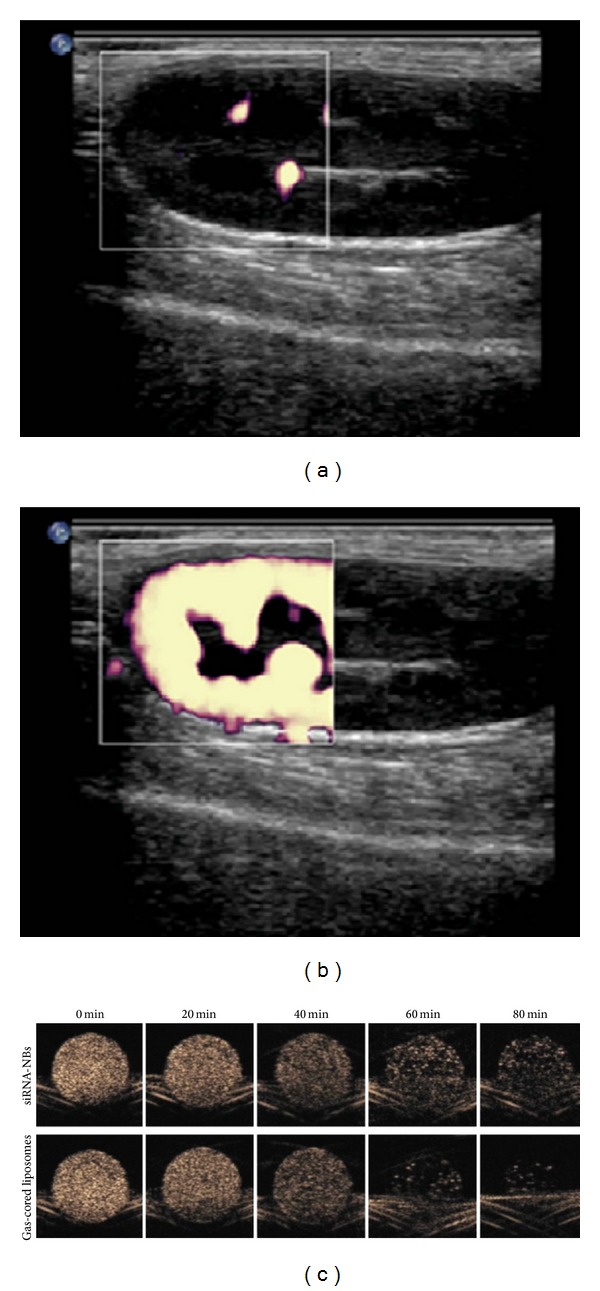
PDI images of New Zealand rabbit kidney. (a) The image was black before the intravenous injection of nanobubbles in rabbit. (b) After intravenous injection of the nanobubbles, PDI enhancement was observed. (c) In vitro contrast enhanced US imaging showed the gray-scale intensities of siRNA-NBs decreased more slowly than the gas-cored liposomes [47, 48].

The nanoscaled ultrasound contrast agent (UCA) can also be used as a theranostic agent with good imaging ability. PLGA nanobubbles show good stability, high-efficiency coating, stable loading, small size, and controlled and efficiency release. Wheatley et al. [49] developed a surfactant-stabilized UCA by differential centrifugation method at a speed of 300 rpm for 3 min. The UCA had an average diameter of 450 nm, which gave 25.5 dB enhancements in vitro at a dose of 10 microL/mL (with a half-life of 13 min). Moreover, the UCA produced wonderful in vivo power Doppler images and grey-scale pulse inversion harmonic images at low sound power levels. Xing et al. [47] fabricated a new kind of biocompatible nanobubbles by ultrasonication of a mixture of polyoxyethylene 40 stearate (PEG 40S) and Span 60 followed by differential centrifugation method. The nanobubbles had a precisely controlled mean size which was small enough to permeate through tumor cell membrane. The differential centrifugation method was an effective method for size separation of particles. It produced narrow size distributions for certain applications. Under the protection of perfluoropropane gas, the bubbles remained stable for more than two weeks. The acoustic behavior of the nanosized contrast agent was evaluated using power Doppler imaging in a normal rabbit model. An excellent power Doppler enhancement was found in vivo renal imaging after intravenous injection of the obtained nanobubbles. The figure showed an example of the reflectivity enhancement by comparing two images, at the beginning of the injection and at the maximum enhancement after injection, respectively. The image appeared black due to no nanobubbles (Figure 2(a)); however, when the nanobubbles were injected in rabbit, marked and complete power Doppler enhancement appeared immediately following slow infusion of the contrast agent and color flare appeared in the renal parenchyma (Figure 2(b)). In vivo power Doppler imaging (PDI) enhancement was observed for about 1 min, suggesting such nanobubbles were stable enough for ultrasound imaging. At the condition of 20 g sample (for 5 min), the maximum enhancement was not observed in PDI modes. This was most likely because of differences in the concentration and stability of the nanobubbles. The imaging observation along with the precipitations for 5 min samples assuredly pointed to better stability for the 3 min samples. According to the experiments, the 3 min and 20 g sample seemed to be the most promising choice for tumor imaging and US-mediated targeted therapy. Yin et al. [48] developed the US-sensitive siRNA-nanobubbles (NBs, referred to as gas-cored liposomes) for tumor imaging and targeted drug delivery. Effective accumulation of the nanobubbles in tumor tissues could be achieved via the EPR effect. The changes of gray-scale intensities before and after US exposure showed that the siRNA-NBs had good US sensitivity, which hold great potential for US-mediated in vivo therapy for tumors. According to the further results, the gray-scale intensities of siRNA-NBs decreased more slowly than the gas-cored liposomes (Figure 2(c)), suggesting good stability; moreover, low-frequency US triggered similarly prompt decrease in gray-scale intensity for both the siRNA-NBs and the liposomes, suggesting that siRNA micelle adhering to liposome surfaces did not alter the sensitivity of the liposomes to ultrasound. With the aid of low-frequency US exposure, siRNA micelles were released from the siRNA-NBs and delivered into tumor cells. Wang et al. [3] used coumarin as a model drug loaded into nanobubbles to investigate the drug delivery potential to cells. The results showed that the nanobubbles (composed of 1% of Tween 80, 3 mg/mL of lipid) presented best in vivo imaging of liver.

Cavalli et al. [50] reported the generation of novel, small-sized, positively charged chitosan nanobubbles. These nanobubbles show the ability to complex with and protect DNA. Their capacity to transfect DNA in vitro was triggered by ultrasound. In the absence of ultrasound, none of the tested DNA-loaded nanobubble concentrations showed any transfection ability. Following 30 seconds of ultrasound treatment, a moderate transfection level was obtained. Shorter sonication times did not result in successful transfection of the DNA cargo into cells, while prolonged sonication times affected cell viability under these test conditions. No formulation-induced cytotoxicity was observed for any of the transfection doses used. This chitosan nanobubble can be considered as an interesting tool in the development of ultrasound-responsive formulations for targeting DNA delivery.

### 4.3. Perfluorocarbon Nanoemulsions

**Figure 3:**
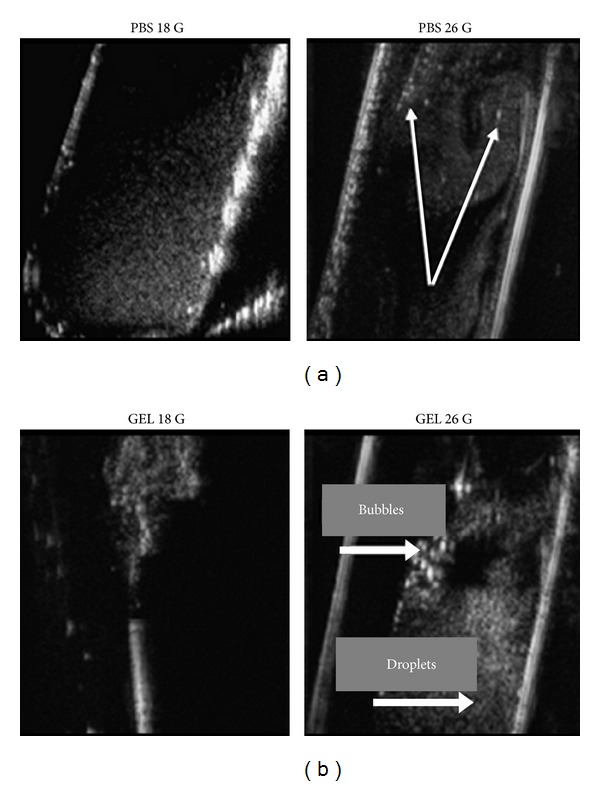
Injection-induced droplet-to-bubble transition. (a) Nanodroplets inserted in PBS through an 18 G needle or 26 G needle. Bubbles formed when nanoemulsion was injected through a thin needle are seen as bright spots (indicated by arrows in the right panel); bubbles rise to the surface while droplets precipitate to the bottom of a test tube. (b) Nanodroplets injected in the agarose gel 18 G (left) or 26 G (right) needles. Injection through the 18 G needle leads to very bright bubbles instantly, whose brightness and size increase over time; the increased brightness of the droplets with time suggesting a droplet-to-bubble transition [53].

The family of liquid perfluorocarbons (PFCs) includes Perfluorodecalin (PFD), Perfluorooctyl bromide (PFOB), Perfluorohexane (PFH), Perfluoropentane (PFP), Perfluorotributylamine (PFTBA), and Perfluoro-15-crown-5-ether (PFCE). PFCs are fluorinated compounds that have been used for many years in clinics mainly as gas/oxygen carriers and for liquid ventilation. Besides this main application, PFCs have also been tested as contrast agents for ultrasonography and magnetic resonance imaging and targeted therapy [51]. A PFC nanoemulsion is prepared by the mixture of perfluorinated hexane and perfluorinated pentane. The nanoemulsion can be prepared by the self-assembly property of polymer and solvent replacement technology. The use of polymer materials wrapping liquid halothane (such as PFP) is a new research direction for preparing nanoemulsions. Under the effect of low-frequency ultrasound, PFH used as the core of phase-change ultrasonic molecular probe has great potential to be an ideal multifunctional agent. PFC particles can infiltrate into arterial walls after balloon injury, cross the internal elastic lamina, and bind and localize molecular epitopes in intramural tissues. Similar PFC nanoparticles targeted to markers of angiogenesis had been successfully used to detect neovasculature around tumors implanted in athymic nude mice using a research ultrasound scanner [52]. Rapoport et al. [53] prepared paclitaxel-loaded perfluorocarbon nanoemulsions stabilized by biodegradable amphiphilic block copolymers, which were systemically injected into mouse models, leading to efficient tumor regression in pancreatic, ovarian, and breast cancer models under the action of ultrasound (1 MHz). Block-copolymer shells of nanoemulsions provide for good in vivo stability and allow enhanced accumulation in the tumor via the EPR effect and the possible active targeting. The drug-loaded perfluorocarbon nanoemulsions could convert into microbubbles locally under the action of ultrasound, resulting in a 125-fold increase of volume and a 25-fold increase of surface area. This in turn resulted in a 25-fold decrease of the primary thickness of the shell. This significantly increased the surface area of copolymer molecule. The droplet-to-bubble transition and bubble oscillation induced drug release and enhanced intracellular uptake. Stable cavitation of microbubbles might be the main mechanism of enhanced drug delivery (Figure 3).

However, the droplet-to-bubble transition is uncontrollable and irreversible. Replacing the PFP with perfluoro-15-crown-5-ether (PFCE, boiling temperature of 146°C) as the core showed a good curative effect in breast and pancreatic cancer animal models [58]. Thakkar et al. [59] developed a perfluorocarbon nanoemulsion by the combination of PFCE and the stable poly (ethylene oxide)-co-poly (DL-lactide) block copolymer shells, which could enhance the permeability of blood vessels upon ultrasound irradiation. And the effect of continuous wave ultrasound was dramatically stronger than that of pulsed ultrasound of the same total energy. PFP nanoemulsion was unstable for storage, and the droplet-to-bubble transition was irreversible. Thus, PFCE core was used to form compound in the second generation of block copolymer stabilized perfluorocarbon nanoemulsions. Passive accumulation in tissue can be enhanced by the traits of nanoscaled size (200 nm to 350 nm) and long circulation of the nanodroplets [58]. Mohan et al. [60] also successfully prepared adriamycin-loaded nanoemulsions for cancer therapy.

### 4.4. Liposomal Nanocarriers

Liposomes (lipid bilayer vesicles) are colloidal structures which can be formed by a mixture of phospholipid and cholesterol in water solution. The internal aqueous pool is formed by self-assembly amphiphilic lipid molecules in solution [61]. Phosphatidylcholine, as the major component of the bilayer lipidic membrane, consists of a natural phospholipids and a phosphate group linked to the hydrophobic section. The film hydration method is a commonly used method in the preparation of liposomes: Various components are typically combined by co-dissolving the lipid in an organic solvent, and then the organic solvent is removed by film deposition under vacuum. When all the solvent is removed, the solid lipid mixture is hydrated by using aqueous buffer. The lipids immediately swell to form liposomes. The conventional lipid film hydration technique has a longer duration of action than the conventional topical formulation [62]. Malheiros et al. [63] developed the liposomes containing the antimicrobial peptide Nisin by reversed-phase, hydration film using probe-type, and bath-type ultrasound. Liposomes are proved to be effective drug carriers, which can carry drugs successfully. Its multifunctional features can be obtained by changing the lipidic membrane composition. Liposomes accumulated in local can significantly improve the efficiency of drug delivery. Liposomes have low immunogenicity, good biocompatibility, and degradability and are often used as the shell of nanobubbles. Compared with polymer-coated materials, liposomal nanocarriers are better in enhancing imaging signal intensity. Piao et al. [64] prepared HSA-LNPs-siRNA (human serum albumin-coated lipid nanoparticles (HSA-LNPs) loaded with phrGFP-targeted siRNA. Their research results showed cell fluorescence and phrGFP mRNA expression were significantly downregulated by HSA-LNPs-siRNA in phrGFP-transfected MCF-7, MDA-MB-231, and SK-BR-3 cells in comparison with control or HSA-LNPs-siRNA (scrambled). In phrGFP-transfected MCF-7 xenograft tumor model, tumor fluorescence was significantly decreased after IV administrations of HSA-LNPs-siRNA at a dose of 3 mg/kg in comparison with siRNA alone. HSA-LNPs-siRNA demonstrated a superior pharmacokinetic profile in comparison with siRNA at a dose of 1 mg/kg. Moreover, no significant cytotoxicity was seen both in vitro and in vivo test. These results show that the novel nonviral carrier, HSA-LNPs, may be used for the delivery of siRNA to breast cancer cells.

**Figure 4:**
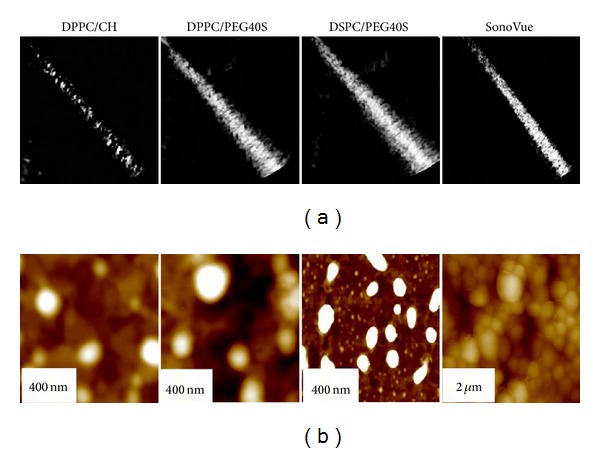
(a) The ultrasound reflectivity of the new lipid formulations and SonoVue. Compared with the commercially available contrast agent SonoVue, the nanoscaled ultrasound active lipid dispersions showed good ultrasound reflectivity. (b) Visualization of diameters by atomic force microscopy [54, 55].

In recent years, it has been reported that ultrasound could effectively control the release of drug from liposomes. UCAs have been reported as therapeutic agents for targeted or controlled drug/gene release. Marxer et al. [54] developed a new kind of drug carriers with an average particle size of 200–300 nm based on different lipid formulations (DPPC/CH, DPPC/PEG40S, DSPC/PEG40S). Compared with the commercially available contrast agent SonoVue, the carriers exhibited adjustable properties such as small size, biocompatibility, good ultrasound reflectivity, high loading capacity, and long circulation (Figure 4(a)). Becker et al. [55] investigated the ultrasound-enhanced thrombolytic effects of the different lipid dispersions (DPPC/CH, DPPC/PEG40S, DSPC/PEG40S, and the SonoVue) in human blood clots. These lipid dispersions showed a mean diameter of about 200 nm by atomic force microscopy (Figure 4(b)). In vitro studies showed that the nanoscaled DSPC/PEG40S dispersion had a best effect on thrombolysis under the action of ultrasound, even without thrombolytic drugs. Stable cavitation was an important fact in fragmenting thrombus.

**Figure 5:**
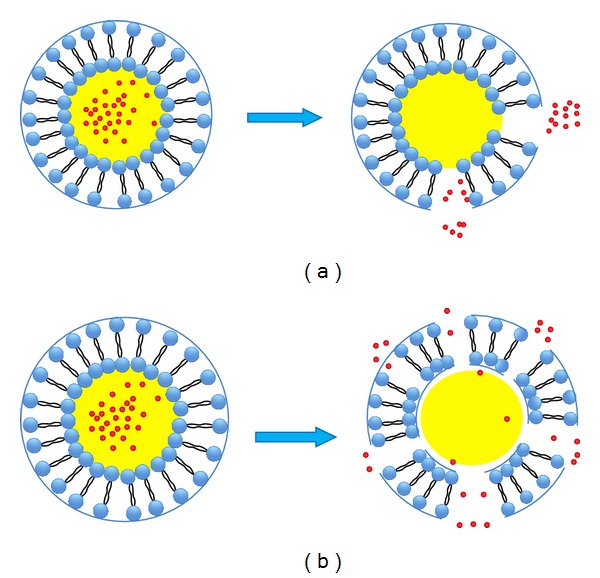
Ultrasound-mediated drug release from eLiposomes. (a) Under the action of low-pressure ultrasound, the droplet vaporizes and expands, breaking the bilayer membrane and leading to release of the contents; this expansion stretches and tears the bilayer membrane (b) or results in cracking into small pieces [56].

The eLiposomes (liposomes which contain emulsion droplets) with lipid bilayer membrane composed of DPPC are more responsive to ultrasound. Lattin et al. [56] developed a kind of eLiposomes by folding interdigitated lipid sheets into closed vesicles around emulsion droplets. The eLiposomes showed excellent sequestration both in the absence of ultrasound and in the presence of low-intensity ultrasound (Figure 5). Further studies showed that the eLiposomes released several times more of the encapsulated calcein than did controls when exposured to 20-kHz ultrasound. Calcein release increased with the exposure time and intensity of ultrasound. The calcein release from the eLiposomes with large (400 nm) droplets was more than that with small (100 nm) droplets, and the PFC6 was not as efficient as the PFC5 in activating calcein release. These observations suggested the use of large PFC5 emulsions in eLiposomes, but the need to construct eLiposomes small enough to extravasate suggested that an optimized intermediate size would be most clinically relevant for drug delivery applications to tumors exhibiting the EPR effect.

**Figure 6:**
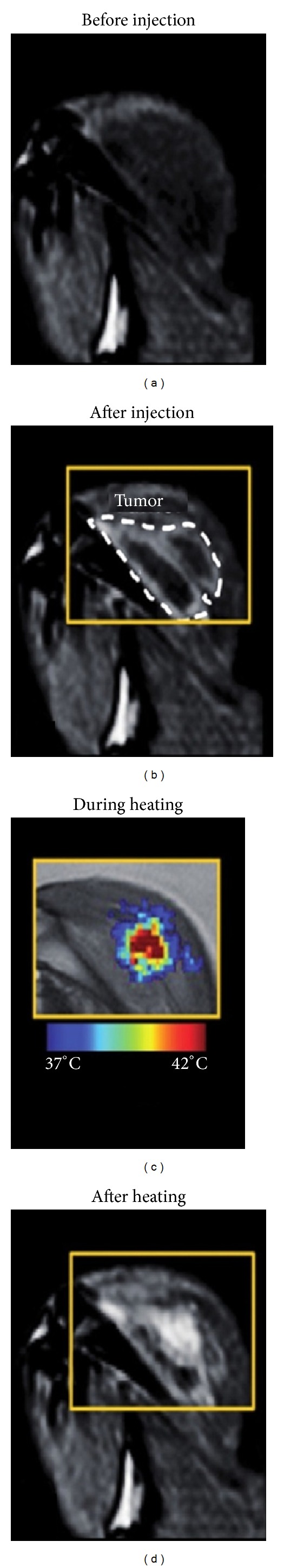
MR signal intensity before and after iLTSL injection and heating with MR-HIFU. Signal intensity: (a) before iLTSL injection and (b) after iLTSL injection. (c) Example of temperature map during heating, overlaid on signal intensity obtained with a treatment planning proton density weighted scan. (d) Signal intensity after four 10 min heating sessions. Note that (a), (b), and (d) represent T1-weighted images, and (c) shows a proton density weighted image [57].

At melting phase-transition temperature, the lipid bilayer membrane changed from gel to the liquid crystalline phase, leading to release of encapsulated drugs from thermosensitive liposomes [65]. Temperature-triggered drug release from a temperature-sensitive liposome (iLTSLs) can promote drug delivery into the cytosol, which may due to the HIFU-induced sonoporation of the cell membrane [66]. MR-HIFU was clinically applied for the treatment of disease, such as uterine fibroids [67], hyperthermia treatments (40–45°C) [57], and hyperthermia-triggered drug delivery in a preclinical setting of rabbits [57, 68]. Based on iLTSLs, release with MR-HIFU was examined in tissue-mimicking phantoms containing iLTSL and in a VX2 rabbit tumor model [57]. In the studies, the in vitro DOX release kinetic of iLTSLs and temperature-induced DOX delivery under MR image-guidance using a clinical MR-HIFU system were studied in great detail [57, 32]. The release of content could be supervised by MRI. Preliminary study showed iLTSL injection could increase MR signal intensity, followed by further increases after each 10 min hyperthermia treatment (Figure 6), which was presumably due to contrast agent release from iLTSL. Thermosensitive liposomes were first used in tumors. Recently, thermosensitive liposomes have been used in other areas. US-mediated delivery using thermosensitive liposomes is helpful to improve the efficiency of drug delivery and has a good application prospect.

### 4.5. Micelles

A micelle is an assembly of amphiphilic surfactant molecules that spontaneously aggregate in water, forming a spherical vesicle. The core of the micelle is hydrophobic and can seclude hydrophobic drugs until released. The micelle is decided by the molecular size and other geometrical characteristics of the surfactants. Polymeric micelles consisting of poly (ethylene oxide)-b-poly (propylene oxide), poly (ethylene oxide)-b-poly (ester), and poly (ethylene oxide)-b-poly (amino acid) hold great promise for drug delivery. Ultrasound-mediate drug delivery with polymeric micelles has been found effective in vivo for treatment of tumors. Via shear stress and shock waves, ultrasound can promote drug release from micelles, which has enormous potential because of fair noninvasion [69]. Nanomicelles can also be used as a stimulation-sensitive drug carrier [70], including pH sensitive (tumor pH, nuclear endoplasm, and lysosome pH), temperature sensitive, and ultrasound sensitive polymer micelle. Polymer micelle can also be modified with ligands, monoclonal antibody, and oligopeptide (mediated across a membrane). Diaz de la Rosa et al. [71] prepared drug-loaded nanomicelles with a diameter of about 10 nm. Husseini et al. [10] prepared the drug-loaded nanomicelles by co-incubating anti-tumor drugs and nanomicelles, which could significantly reduce the side effects of chemotherapy drugs. The nanomicelle is a good indicator of ultrasound-mediated drug delivery system with a low threshold. Polymeric micelles such as PEO-PPO block copolymers are safer and kinetically stable and have higher solubilization capacity than the regular micelles [72, 73]. Despite all these advantages, PEO-PPO block copolymers still present a number of limitations such as low stability and short residence times, which limit their wide application [74]. Diaz de la Rosa et al. [71] reported that drug could release from nanoscaled micelle at 70 KHz, but not at 500 KHz; moreover, inertial bubble collapse was not a sufficient requirement for drug release at either frequency. Therefore, the biological effects produced more intensely.

### 4.6. Albumin Nanoparticles

Albumin, an excellent carrier, can be used for drug/gene delivery. The albumin nanoparticles measured by the method of dynamic light scattering are approximately 100 nm in diameter [76]. It is very promising for being nontoxic, nonimmunogenic, high biocompatible, and easy biodegradable. Albumin nanoparticles coupled with targeting ligands presented high drug loading capacity. Specialized nanotechniques such as emulsification, thermal gelation desolvation, nanospray drying, and self-assembly have been used for manufacture of albumin nanoparticles. Albumin nanoparticles also have gained considerable attention for the high load capacity of drugs/genes. Moreover, albumin nanoparticles have almost no side effects. Site-specific drug targeting refers to a variety of ligands applied to modify the surface of albumin. Apolipoprotein E was advised to mediate the drug traversing through the blood brain barrier. Michaelis et al. [77] designed and developed human serum albumin nanoparticles (HSA-NP) using apolipoprotein E nanoparticles, which could cross the blood brain barrier with loperamide as model drug to exert antinociceptive effects. Apolipoprotein E was associated with loperamide-loaded HSA-NP by chemical method. Antinociceptive effect in ICR mice after intravenous injection showed that the nanoparticles enhanced drugs across the blood brain barrier.

**Figure 7:**
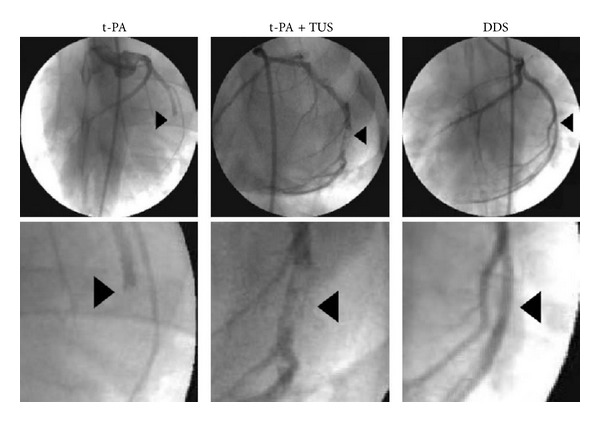
Coronary angiography after thrombolysis. Typical coronary angiographic images at 60 min in swine treated with t-PA (55,000 IU/kg) alone, t-PA plus TUS, and DDS. The lower images are enlargements of each affected site. Arrowheads indicate the site of thrombotic occlusion before treatment [75].

There are some exciting clinical applications of albumin, such as photodynamic therapy, transport protein for metal complexes, and an anti-HIV agent. Albumin bubbles can burst and release the drug after destruction by ultrasound. It can also be used as an artificial blood substitute with the development of tetraphenylporphyrinato-iron (II) bound to albumin. Human serum albumin (HSA) together with polyethylenimine (PEI) was formed as a nonviral gene delivery vehicle and tested for transfection efficiency in vitro. Spectrophotometric analysis was used to determine the stability and transfection efficiency was evaluated in cell culture using human embryonic epithelial kidney 293 cells. Optimal transfection efficiency was obtained when the particles were prepared at N/P ratios between 4.8 and 8.4. Kawata et al. [75] designed a novel nanosized delivery system of tissue-type plasminogen activator (t-PA) as a therapy to coronary thrombolysis; the results showed it had a suppressed thrombolytic activity of t-PA in acute myocardial infarction model after injecting of t-PA nanoparticles (25% t-PA, 55000 iu/kg) and would not increase the risk of bleeding but recovered the activity only under the action of ultrasound (1.0 MHz, 1.0 W/cm^2^) (Figure 7).

With the development of nano-controlled release technology, ultrasound-mediated intelligent drug delivery system (DDS) has great prospect for the development of nanoscaled drug delivery system and acoustic trigger system. The system is mainly composed of the t-PA gene, basic gelatin, zinc ions (restrain activity of t-PA), and in vitro ultrasound. The ultrasound-mediated recovery of t-PA activity synergistically promotes the thrombolytic activity.

## 5. Conclusion 

In summary, the combination of nanocarriers and ultrasonic irradiation has great potential for diagnosis and treatment of disease. Ultrasound can facilitate the transport of drug/gene by increasing vascular and cellular permeability, and nanocarriers can accumulate in pathological tissues via the enhanced EPR effect. The system can increase the therapeutic effect and reduce adverse reactions. Therefore, it is expected that this new technology will be utilized as a novel delivery method in clinical field. However, although the combination of nanocarriers and ultrasonic irradiation has a lot of advantages for drug/gene delivery and targeted therapy, there are many problems and difficulties that need to be solved: (a) the key preparation technology of nanocarriers needs to be further optimized; (b) the construction process of targeted nanocarriers is tedious, so production methods need to be improved; (c) to find ligands coupled with the bubble can actively identify tumor-specific markers; antibody is an ideal molecule for the construction of targeting US with small volume, good affinity for antigen molecules, high soluble, thermoresistance, and stronger resistance to acid and alkali. Multifunctional nanocarriers combining a specific targeting agent (usually an antibody or peptide) should be developed; (d) ultrasound can increase penetrability of cell membrane, while the relatively higher concentration of nanocarriers and higher strength may lead to damages of the surrounding normal cells and tissues. It therefore seems reasonable to assume that nanocarriers, in combination with diagnostic, therapeutic, and theranostic US, will gain ever more importance in the years to come, both at the preclinical level and in patients. The results also encourage further investigation of the possible diagnostic and therapeutic benefits of using nanoparticles as carriers, including passive targeting and accumulation in tumors. Further research will lead to the creation of intelligent/smart particles, for example, thermosensitive particles which release the active ingredients at a specific temperature.
